# Perspective on long term oxygen therapy

**DOI:** 10.1186/2049-6958-7-16

**Published:** 2012-07-17

**Authors:** Peter Howard

**Affiliations:** 1 Former Reader in Medicine, University of Sheffield, 3 Sandygate Park, Sheffield, UK

## 

The paper by Dal Negro *et al.*[1] raises a number of interesting issues. Patients with severe chronic obstructive pulmonary disease (COPD) were ranked according to hemoglobin in a program of monitored long term domiciliary oxygen therapy (LTOT). Good therapeutic compliance (mean 17 hours/24 hour day) using liquid oxygen at a flow rate of 2.3 L per minute, presumably through nasal cannulae, was achieved. This is remarkable: few schemes achieve such good adherence. Patients were mostly male, ex-smokers of mean age 71.4 years, of average body weight with few markedly over- or underweight.

Blood hemoglobin ranged from moderately anaemic levels to frankly polycythemic. Anemic patients tended to be under weight raising the issues, as the authors discuss, of the consequences of systemic inflammatory disease, in some cases of advanced emphysematous COPD.

The most interesting finding in this small study was the striking improvement not only in PaO_2_ but in PaCO_2_ with a trend to continuing benefit throughout the period. Similarly, hemoglobin crept in from the extreme limits to a normal middle range. Whilst it would not be surprising for polycythemia to redress, the idea that systemic inflammation might be reversible, indicated by a rising hemoglobin in response to LTOT in advanced COPD with emphysema, is notable.

It is approaching 40 years since the original domiciliary oxygen therapy trials [2,3] were conducted in the UK and USA. It is worth comparing the type of patients enrolled in those trials with those treated today. In the UK, the mean age of the patients was in the early 50’s compared with over 70 years today. They suffered advanced COPD, were generally overweight, very edematous, polycythemic with hypoxemia and hypercapnia, the latter often severe. Hypercapnia limited the application of supplementary oxygen by its increase under oxygen administration. There was an increase in survival in the oxygen treated group through relief of extreme hypoxemia with no evidence to suggest attenuation or improvement of progressive COPD. The net result was an extension of life for two to three years.

Since then, the natural history of COPD has changed. The time course of the pathological features has extended. Whilst death from COPD remains a major health issue, it is now a disorder predominantly of the over-70 years of age. Respiratory failure develops some 20–30 years later than it used to.

Is it the same disease? There is now less extreme hypercapnia [4], obesity, edema, polycythemia but more underweight, extreme dyspnea, anemia and systemic markers of inflammation. It is still considered an inevitably progressive disorder for which current therapies provide amelioration rather than reversal of the destructive processes.

This paper raises the question as to whether there is a therapeutic window during the now prolonged natural history to end-stage disease and death when therapeutic interventions such as well controlled LTOT might further attenuate outcome. This study is too small to reply to this question, but provides a challenge to the authors to engage in a much bigger, probably multi-center, study.
